# Supplementary material for: The interactome of intact mitochondria by cross-linking mass spectrometry provides evidence for coexisting respiratory supercomplexes
Source: Mol Cell Proteomics. 2017 Dec 8;17(2):216–32. doi: 10.1074/mcp.RA117.000470 (PMC5795388; doi:10.1074/mcp.RA117.000470)
Supplement: Supplemental Data [file supp_RA117.000470_133922_0_supp_23978_fzffwf.zip › spectra_annotation/mito_DR_spectra_annotation/251-1-1-1-8-1.pdf]

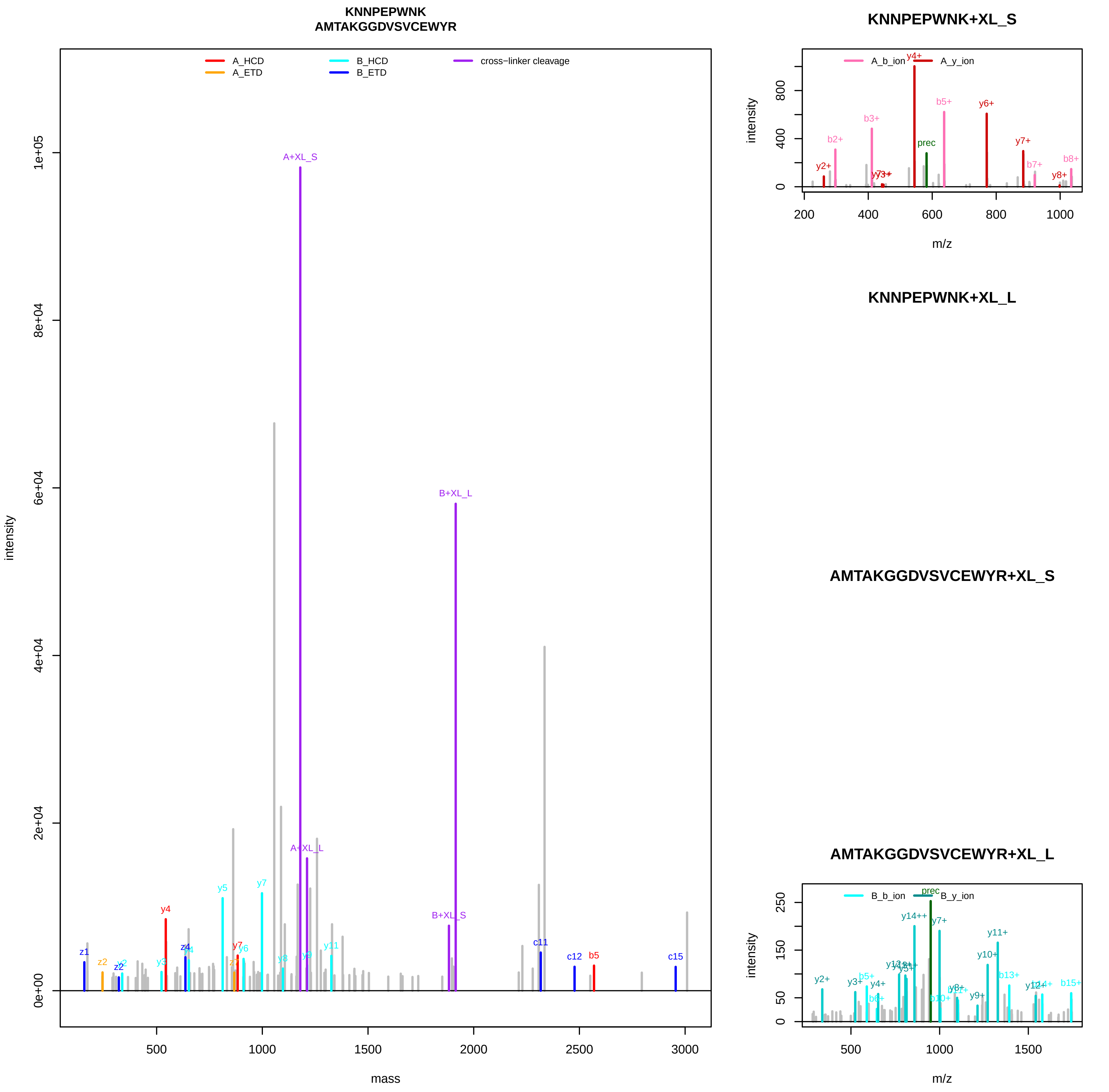
KNNPEPWNK
AMTAKGGDVSVCEWYR
A_HCD
A_ETD
B_HCD
B_ETD
cross−linker cleavage
A+XL_S
B+XL_L
A+XL_L
B+XL_S
intensity
mass
0e+00
2e+04
4e+04
6e+04
8e+04
1e+05
500
1000
1500
2000
2500
3000
z1
z2
z4
y4
y5
y7
y6
y11
c11
c12
b5
c15
KNNPEPWNK+XL_S
A_b_ion
A_y_ion
y4+
b5+
y6+
b3+
b2+
prec
y7+
b8+
y2+
b7+
y8+
m/z
KNNPEPWNK+XL_L
AMTAKGGDVSVCEWYR+XL_S
AMTAKGGDVSVCEWYR+XL_L
B_b_ion
B_y_ion
y14++
y7+
y11+
y10+
y2+
y3+
b5+
y4+
b6+
b10+
y9+
b13+
y12+
b15+
